# Allogeneic tumor cell line-based vaccines: A good alternative to autologous and cancer stem cell vaccines in colorectal cancer

**DOI:** 10.22038/ijbms.2021.56732.12671

**Published:** 2021-09

**Authors:** Fatemeh Rafieenia, Elham Nikkhah, Fatemeh Nourmohammadi, Susan Hosseini, Abbas Abdollahi, Nurieh Sharifi, Mohsen Aliakbarian, Mohammad Mahdi Forghani Fard, Mehran Gholamin, Mohammad Reza Abbaszadegan

**Affiliations:** 1 Medical Genetics Research Center, Mashhad University of Medical Sciences, Mashhad, Iran; 2 Department of Pharmacodynamics and Toxicology, School of Pharmacy, Mashhad University of Medical Sciences, Mashhad, Iran; 3 Department of Biology, Damghan branch, Islamic Azad University, Damghan, Iran; 4 Surgical Oncology Research Center, Mashhad University of Medical Sciences, Mashhad, Iran; 5 Department of Pathology, School of Medicine, Mashhad University of Medical Sciences, Mashhad, Iran; 6 Department of Laboratory Sciences, School of Paramedical Sciences, Mashhad University of Medical Sciences, Mashhad, Iran; 7 Immunology Research Center, Mashhad University of Medical Sciences, Mashhad, Iran

**Keywords:** Allogeneic, Autologous, Cancer stem cell, Colorectal cancer, Vaccine

## Abstract

**Objective(s):**

Besides the uncertainty about colorectal cancer stem cell (CCSC) markers, isolating, purifying, and enriching CCSCs to produce CCSC vaccines is highly challenging. However, allogeneic vaccines developed from CRC cell lines can provide universal, comprehensive, inexpensive, simple, and fast approach to cancer treatment.

**Materials and Methods:**

CCSCs were isolated from human CRC tissue using the in vitro sphere formation assay and then characterized through gene expression analysis, in vivo and in vitro tumor formation assay, karyotyping, and surface marker detection. Subsequently, CCSCs and two CRC cell lines (HT-29 and SW-480) were inactivated with cisplatin (CDDP) and administrated as vaccines to the three groups of athymic C57BL/6 nude mice. Afterward, tumorigenesis was challenged with HT-29 cells. The antitumor effect of vaccines was evaluated by tumor and spleen examination and immune response analysis. The cytotoxic activity of splenocytes and serum levels of TGF-β and IFN-γ were measured by Calcein-AM cytotoxicity assay and enzyme-linked immunosorbent assay (ELISA), respectively.

**Results:**

The results of gene expression analysis showed that CCSCs are CD44+CD133-LGR5-. All vaccinations resulted in decreased tumor growth, spleen enlargement, enhanced serum level of IFN-γ and TGF-β, and increased cytotoxic activity of natural killer (NK) cells. The antitumor efficacy of the CCSC vaccine was not more than CRC cell line-based vaccines. Interestingly, the allogeneic SW-480 vaccine could effectively inhibit tumorigenesis.

**Conclusion:**

Despite the great challenge in developing CCSC vaccines, allogeneic vaccines based on CRC cell lines can efficiently induce antitumor immunity in CRC.

## Introduction

**Figure 1:**
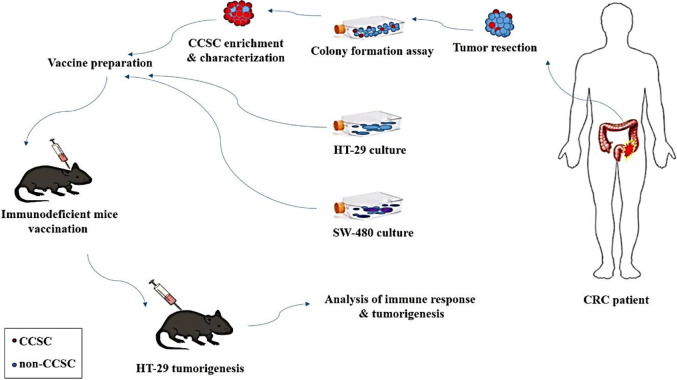
The schematic diagram of the experimental design. After isolation and characterization of CCSCs from the CRC patient, the isolated CCSCs as well as two CRC cell lines (HT-29 and SW-480) were inactivated with CCDP and injected subcutaneously into the necks of nude mice in three stages. 10 days after vaccination, tumorigenesis was challenged with HT-29 cells in the right flank

According to the global cancer observatory in 2018, colorectal cancer (CRC) is the second most frequent cause of cancer-related death in the world (1) which is increasing among young people (2). Every year, many cancer patients die not from CRC but due to the side effects of routine treatments such as surgery, chemotherapy, and radiation therapy. Also, inadequacy and lack of specificity of these methods often lead to cancer resistance and recurrence. Over the past decades, accumulating discoveries about immunotherapy have provided new potential strategies for cancer treatment. Adoptive cell therapy and cancer vaccines are the two major immunotherapeutic approaches in CRC. Cancer vaccines include dendritic cell vaccines, tumor cell vaccines, DNA vaccines, peptide vaccines, and viral vector-based vaccines (3). Unlike other immunotherapeutic strategies, tumor cell vaccines stimulate the immune system against a wide range of tumor-associated antigens (TAAs). They can also be used for all cancer patients, regardless of their HLA type (4). Given the uncertain nature of cancer antigens, whole tumor cells appear to be an appropriate and feasible vaccine development option. Depending on antigen source, tumor cell vaccines could be autologous or allogeneic type, including OncoVAX (5) and GVAX (6), respectively. GVAX is composed of whole tumor cells genetically modified to secrete the immune-stimulatory cytokine, granulocyte-macrophage colony-stimulating factor (GM-CSF), and irradiated to prevent further cell division (7). Allogeneic tumor cell vaccines are being widely studied in clinical trials. The GVAX colon vaccine is developed from the two CRC cell lines SW-837 and SW-620. Another allogeneic tumor cell vaccine, CancerVax, consists of three human melanoma cell lines, including a wide range of TAAs and major histocompatibility complex antigens (6, 8, 9). A CRC vaccine composed of irradiated, allogeneic human CRC cell lines along with GM-CSF-producing bystander cells and cyclophosphamide in a phase 1 study showed safety and feasibility in patients with metastatic CRC (10). However, researchers are extensively looking for targeted therapy against cancer stem cells (CSCs). CSCs are a small population of tumor cells with slow proliferation and are responsible for tumor initiation and recurrence (11). However, providing the CSC vaccines is successful or practical only in a small group of patients. On the other hand, tumor heterogeneity and cancer cell plasticity have made CSC markers a challenge in CSC vaccines. Allogeneic vaccines developed from CRC cell lines have significant advantages over autologous or CSC vaccines in terms of availability, easy and rapid preparation, and possibility of multiple vaccinations. In this study, CCSCs were isolated and characterized from primary CRC tissue. Then, the efficacy of vaccines prepared from CCSCs and human CRC cell lines was investigated in nude mice. The two human CRC cell lines HT-29 and SW-480 were selected as autologous and allogeneic vaccines, respectively. The experiment steps are shown schematically in Figure 1.

## Materials and Methods

**Cell culture and CCSC characterization**

CCSCs were isolated from the CRC patient and then characterized by *in vitro* and *in vivo* analysis. Also, CRC cell lines (HT-29 and SW-480) were cultured for vaccine preparation.

***Patient***

*After explain*ing the purpose of the project, informed consent was obtained from seven participants. The age and sex of the patient as well as the stage, grade, or location of the tumor, chemotherapy and radiotherapy treatments, or family history were not criteria for patient selection. Our criterion was patient consent and then pathological confirmation of the tumor sample. Finally, CCSCs were isolated from the CRC tumor of a 51-year-old man from Ghaem hospital, Mashhad, Iran. The patient had not received any chemotherapy or radiotherapy. The invasive CRC adenocarcinoma with a tumor grade of T3N0MX was pathologically confirmed. Tumor and normal tumor-adjacent epithelial tissues were obtained from rectosigmoid regions directly after surgical removal. 

***CCSC Isolation from CRC tissue***

*After severe rinsing with phosphate-buffered saline (PBS) containing 5X antibiotic cocktail of 250* µg/ml gentamicin, 5% Pen/Strep, and 12.5 ug/ml amphotericin B, fresh tumor tissue was mechanically minced with scissors into 3 mm^3^ pieces. Then, tumor fragments were incubated overnight at 4 °C in DMEM/F12 medium (Gibco, Cat#32500-035) containing 4X Primocin (8 μg/ml) (Invivogene, Cat#ant-pm-1). The day after, tumor fragments were incubated for at least 2 hr in DMEM/F12 medium containing collagenase type IV (200 U/ml) (Gibco, Cat#17104-019) and 6 μg/ml primocin. Single cells passed through 40 μm cell strainers (SPL, Korea) were incubated for 10 min in red blood cell lysis buffer and centrifuged at 1200 RPM for 5 min. Cancer cells were cultured in 6-well ultra-low attachment plates (Corning, Cat#CLS3471) with serum-free DMEM/F12 containing 15 mM HEPES, 10 ng/ml bFGF (Sigma, Cat#SRP4037), 20 ng/ml EGF (Sigma, Cat#E9644), 10 ng/ml LIF (Sigma, Cat#L5283), 2% B-27 supplement (Gibco, Cat# 17504-044), 8 μg/ml primocin at 37 °C and humidified 5% CO_2_ incubator. 

**The characterization of CCSCs**

CCSCs were characterized by tumorigenesis *in vitro *and* in vivo*, the differentiation capacity assay, karyotyping, and surface markers (12-14).

**In vitro tumor formation assay**

The proliferated CCSCs were dissociated into the single cells with TrypLE™ Express Enzyme (Gibco, Cat#12604013). Considering the self-renewal capacity of CCSCs, they were re-cultured in a 6-well ultra-low attachment plate into the serum-free medium to evaluate the regeneration of 3D spheroids of CCSCs (colonospheres).

The differentiation capacity assay

CCSCs could differentiate to other types of cells, including mesenchymal-like cells. Then, singled CCSCs (passage 6) were cultured in DMEM/F12 medium supplemented with 10% inactivated fetal bovine serum (FBS) (Gibco Cat#10270106) in 6-well plates (SPL, Korea).

**In vivo tumor formation assay**

After the approval of the institutional animal care and use committee of Mashhad University of Medical Sciences (MUMS), 3×106 CCSCs (passages 4-10) were subcutaneously injected with 100 µl cold Matrigel (Corning, Cat#356234) in the right flank of 7 to 8-week-old healthy female C57BL/6 nude mice (purchased from North Research Center, Pasteur Institute of Iran). The mouse was in a sterile animal room with a 12-hr light/dark cycle and 40-50% humidity and 22 °C. After two weeks, the mouse was euthanized with an intraperitoneal injection of the mixed solution of ketamine (100 mg/kg) and xylazine (10 mg/kg). The skin of the tumor area was then cut with scissors and forceps and the tumor was removed.

**Detection of surface markers CD44 and CD133 by Flow cytometry**

CCSCs were singled with TrypLE™ and separately incubated on ice for 20 min in darkness with APC anti-mouse/human CD44 (BD Pharmingen, Cat# 559942), and PE anti-human CD133 (Milteny Biotec, Cat# 130-113-670). The labeled cells were then twice washed with PBS and centrifuged for 5 min at 1200 RPM. Fluorescence was measured by a flow cytometer (BD Accuri C6, BD Biosciences, USA), and FlowJo software was used for the data analysis.

**Karyotyping**

The standard protocol was used for chromosomal analysis (15). Metaphase harvesting was carried out on the 25th passage CCSCs. After 15 min incubation with 500 µl colcemid at 37 °C, CCSCs were treated with hypotonic KCl (0.075 M) for 20 min. After fixation with a mixture of methanol and glacial acetic acid (3:1), GBG (G-bands after 5′-bromodeoxyuridine and Giemsa) banding was analyzed by the Cytovision program in 50 metaphases.

***RNA extraction and cDNA synthesis***

Using TRIzol™ Plus RNA purification kit (Invitrogen, Cat#12183555), total RNA was extracted from CCSCs (6th passage) and the adjacent normal tissue. After determining the quantity and quality of mRNAs by a spectrophotometer (Biochrom WPA Biowave DNA Life Science), RNA samples were treated with DNaseI (Fermentas, Cat#EN0521) along with Rnase Inhibitor (Fermentas, Cat#EO0381). cDNAs were synthesized by an Easy cDNA Synthesis Kit (Pars Tous Biotechnology, Mashhad, Iran). 

***Primer design & quantitative real-time PCR analysis***

Gene expression analysis was performed at least three times for some specific and universal CSC markers, including ALDH1A1, LGR5, CD44, CD133, EPCAM, CTNNB1, MYC, SOX2, CDH1, and CDH2 (Table 1). Phosphoglycerate kinase 1 (PGK1) has been reported as one of the most stable CRC genes, even under 3D cultures (16, 17). Therefore, it was used as a reference gene in this study. The SYBR^®^ Premix Ex Taq™ II (Takara, Cat#RR820L), the LightCycler® 96 Instrument (Roche), and the comparative CT method were used for quantitative real-time PCR analysis. Primers were designed using the database https://www.ncbi.nlm.nih.gov/tools/primer-blast/. The real-time PCR was performed at 60 °C for all primer sets.

PGK1: phosphoglycerate kinase 1, ALDH1A1: Aldehyde dehydrogenase 1 family member A1, EPCAM: Epithelial cell adhesion molecule, SOX2: sex-determining region Y-box 2, LGR5: Leucine-rich repeat-containing G-protein coupled receptor 5, CDH1: Cadherin-1, CDH2: Cadherin-2. 

***Cell line culture***

HT-29 and SW-480 were cultured in DMEM/F12 medium supplemented with 10% FBS and 1% Pen/Strep and incubated under conditions of 95% humidity, 5% CO_2_, and 37 °C. 

***Vaccination***

The median lethal concentration (LC_50_) dose of CDDP for vaccine inactivation was determined by 3-[4, 5-dimethylthiazol-2-yl]-2, 5 diphenyl tetrazolium bromide (MTT) assay. After vaccine preparation, inactivated vaccines were injected into C57BL/6 nude mice.

**MTT assay**

Five×10^3^ cells of HT-29, SW-480, and CCSCs were cultured in a 96-well flat-bottom plate (SPL, Korea). After 24 hr incubation at 37 ***°C*** in 5% CO_2_, media was changed with various CDDP concentrations in triplicate and incubated for 2 hr at 37 ***°***C in 5% CO_2_. Then, media was discarded and 100 µl medium containing 0.5 mg/ml MTT (Sigma, Cat# M5655-1g) was added to the wells. After 2 hr incubation at 37 °C in 5% CO_2_, the medium was slowly removed, and 100 µl DMSO was added to each well. The plate was wrapped in foil, shaken on an orbital shaker for 30 min, and read at 570 nm with an ELISA Reader (ELx800, BioTech, USA). The percentage of the viable cells was calculated using the following formula: [(sample abs)/ (control abs) × 100)].

***Mice immunization***

Twenty-four C57BL/6 nude mice (female, 16–22 g, and 6-8 weeks old) were randomly divided into four groups with six mice per group: CCSCs, HT-29, SW-480, and saline. 5×104 CDDP-inactivated cells were injected into the vaccine groups three times at two-week intervals (18) by subcutaneous injection in the neck. The CCSCs were in passages 4-10. Ten days after the last vaccination, all immunized mice were challenged subcutaneously with 3×106 HT-29 cells in the right flank. The endpoint of the study was tumor size of about 20 mm in the saline group. Two weeks after tumorigenesis, mice were euthanized to remove the tumor and spleen and to take blood samples from the heart. Tumor size was calculated using the Monga formula (L×W×H)/2 (19). Mice were monitored daily for general health and body weight and mice with weight loss or scoliosis were excluded. Finally, at least three mice from each group survived to the end of the experiment (two months), which were further analyzed. 

***The immune response***

Fresh blood was obtained from the hearts of mice under anesthesia. Serum levels of TGF-β and IFN-γ were measured using ELISA kits according to the manufacturer’s protocol (TGF-β: Bioassay technology laboratory, Cat# E0660MO, and IFN-γ: Invitrogen, Cat# CN.88-7711-44). Samples were read at 450/570 nm using an ELISA reader (ELx800, BioTech, USA). 

***NK cytotoxicity***

The spleen tissues were harvested from the immunized mice. YAC-1 cells were labeled with 100nM Calcein-AM (Invitrogen, Cat# C3099) at 37 °C in 5% CO_2_ for 20 min in serum-free medium and darkness. Labeled YAC-1 cells were washed three times in PBS containing 5% FBS with a 5 min centrifuge at 1200 RPM. The splenocytes as effector cells were seeded with the labeled YAC-1 target cells in a 96-well plate at 20:1 ratios of effector cells to target cells in triplicate. After 4 hr incubation at 37 °C, the supernatant was separated by centrifuge at 1200 RPM for 5 min. The fluorescence of 100 μl of supernatants was monitored at 485/530 nm excitation/emission wavelengths using Synergy™ H4 Hybrid Multi-Mode Microplate Reader (BioTek, Vermont, USA) in black 96-well flat-bottomed plates (SPL Life Sciences, Gyeonggi-do, Korea). The cytotoxicity of the NK cells was calculated through lysis percentage: [(experimental release− spontaneous release)/ (maximum release− spontaneous release)] × 100. Maximum release and spontaneous release were obtained from incubation of YAC-1 cells with and without 2% Triton X100, respectively (18, 20). 

**Hematoxylin and eosin (H&E) staining of tumors**

After 24 hr incubation in 10% formalin, tumors resected from mice were stained with H&E and then examined pathologically.

**Statistical analysis**

Graphpad Prism (version 8) was used for data analysis. Turkey’s multiple comparisons test was used for comparison between groups. 

## Results

**The characterization of CCSCs**

**Figure 2:**
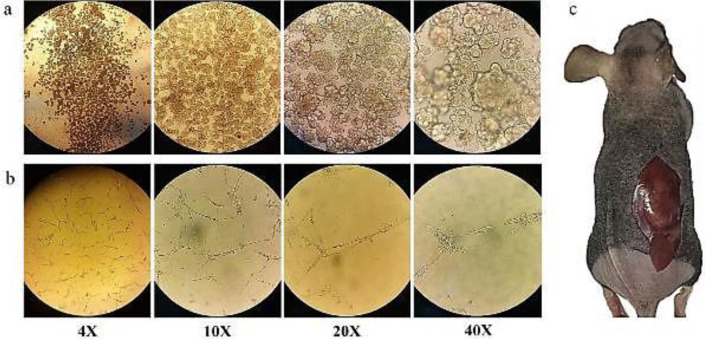
Isolation and proliferation of CCSCs: a) Colonospheres were formed 1-7 days after re-culturing singled CCSCs into ultra-low attachment plate (6th passage). b) CCSCs showed epithelial to mesenchymal transition under adherent culture with serum containing medium (6th passage). c) CCSCs formed tumors in the female C57BL/6 nude mice two weeks after transplantation of ~3×106 cells (passage 4-10) in the right flank

CCSCs could reform colonospheres in the ultra-low attachment plate in the serum-free medium (Figure 2a). The differentiation capacity was confirmed by the epithelial to mesenchymal transition of CCSCs (Figure 2b). The isolated CCSCs could also form tumors in the female C57BL/6 nude mice (Figure 2c). 

***The real-time PCR gene expression analysis***

**Figure 3:**
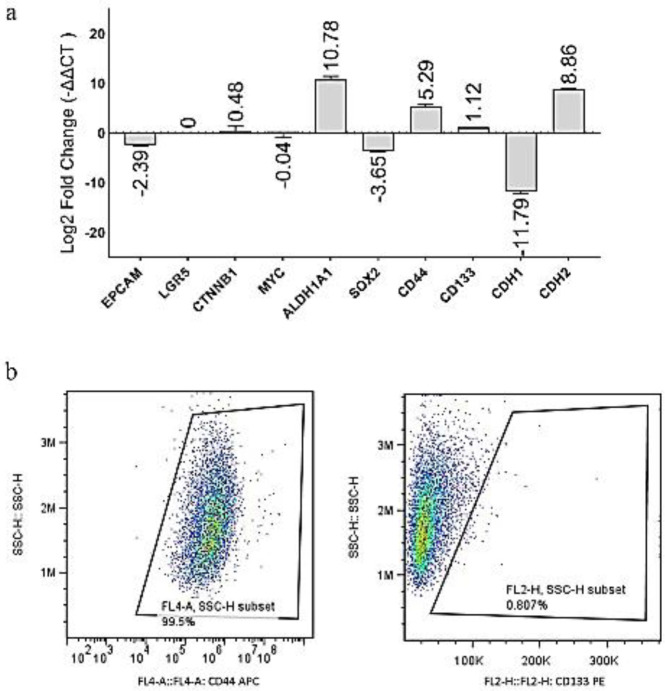
Results of the gene expression analysis and flow cytometry: a) ALDH1A1, CDH2, and CD44 had the highest overexpression in CCSCs, respectively. EPCAM, SOX2, and CDH1 were decreased in CCSCs. The expression of LGR5 was not detected. No significant change (± 2-fold) was observed for CD133, CTNNB1, and MYC. b) The flow cytometry results showed that CD44 was highly expressed in CCSCs, and on the other hand, CCSCs were negative for CD133

***The *** ΔΔCT comparison results (Figure 3a) showed that ALDH1A1 (∽ 2^11^ fold, CDH2 (∽ 2^9^ fold), and CD44 (∽ 2^5^ fold) had the highest overexpression in CCSCs, respectively. LGR5 expression was not observed in the normal tissue or CCSCs. EPCAM (∽ 2^2^ fold), SOX2 (∽ 2^4^ fold), and CDH1 (∽ 2^12^ fold) were decreased in CCSCs. 

**Analysis of the surface markers CD44 and CD133**

CCSCs have been identified as CD44^+^CD133^-^ by flow cytometry (Figure 3b). The frequency of CD44^+^ cells and CD133^+^ cells was 99.5% and 0.8%, respectively. 

**Karyotyping**

**Figure 4:**
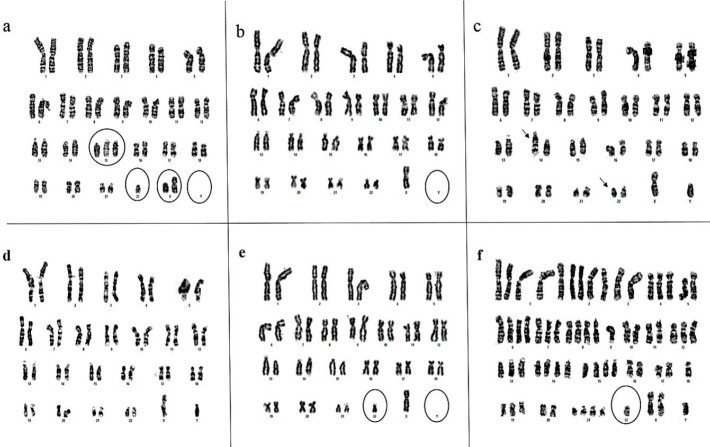
karyotyping results: Analysis of 25^th^ passage CCSCs at 50 metaphases revealed numerical and structural chromosomal abnormalities: a) 46, XY, +15, -22 [9], b) 45, X, -Y [8], c) 46, XY, der (14) t (14;22) (q32; q11) [7], d) 46, XY [8], e) 44, X, -Y, -22 [8], f) 64, XXY, -3, -4, -5, -5, -7, -9, -9, -9, -10, -10, -11, -11, -12, -13, -16, -16, -17, -17, -18, -18, -18, -19, -20, -20, -22, -22, -22 [10]

Karyotyping showed the heterogeneity in CCSCs. The structural and numerical abnormalities were observed in most of the chromosomes, especially Y and 22 (Figure 4). Deletion of chromosome 22 seems to play an effective role in malignancy. In other words, this chromosome has important tumor suppressor genes. The chromosomal range was 44-64.

***The MTT assay***

**Figure 5:**
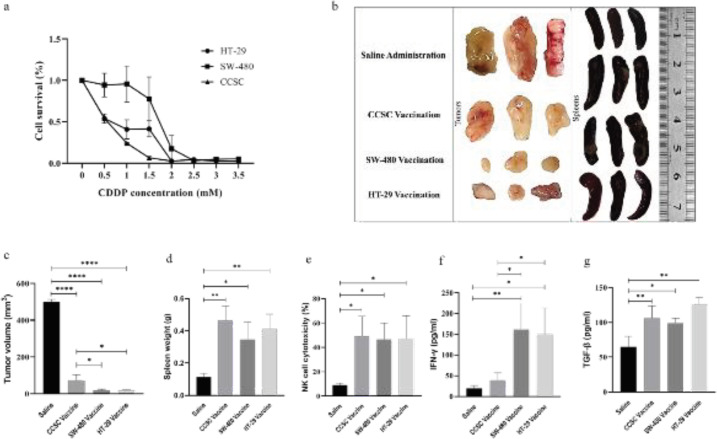
MTT and vaccination results: a) SW-480 was more resistant to CDDP than HT-29 and CCSCs. b) Vaccinations have led to tumor shrinkage and spleen enlargement in all vaccine groups (four groups with three mice in each group). c) All vaccines inhibit tumorigenesis in the vaccinated mice. However, the effect of CCSC vaccination on tumor size was less than that of cell line vaccines. d) Spleen weight was increased in all groups. e) All vaccines increased the activity of splenocytes. f) IFN-γ showed a significant increase in SW-480 and HT-29 vaccine groups but not in the CCSC group. g) TGF-β increased in all vaccine groups. Statistically significant difference is shown by * in *P*<0.05, ** in *P*<0.01, *** in *P*<0.003, and **** in *P*<0.0001

Based on LC_50_ values, CDDP concentrations for vaccine inactivation were gained 0.6mM for CCSCs and HT-29, and 1.7mM for SW-480 (Figure 5a). 

**The effect of vaccination on tumor size**

Figure 5b shows changes in tumor and spleen size after vaccination. All vaccinations inhibited tumorigenesis (Figure 5c) and increased spleen weight (Figure 5d) compared with the control group. The tumor volume in mice vaccinated with the CRC cell lines was statistically significantly decreased (*P*<0.05) compared with the mice immunized with the CCSC vaccine. Similar to the autologous HT-29 vaccine, the allogeneic SW-480 vaccine could effectively inhibit tumorigenesis.

***The NK cytotoxicity assay***

The cytotoxic activity of splenocytes increased in all vaccine groups compared with the control group with P<0.05 (Figure 5e).

***The immune response***

Despite HT-29 and SW-480 vaccine groups, IFN-γ was not significantly increased in the CCSC vaccine group (Figure 5f). IFN-γ was markedly increased in the SW-480 vaccine group compared with the other groups (P<0.01). IFN-γ was increased in cell line-based vaccine groups further than the CCSC vaccine group (P<0.05). TGF-β was significantly increased in all vaccine groups compared with the saline group (Figure 5g). Tumor volume showed a negative correlation with TGF-β (P<0.05), IFN-γ (P<0.01), and NK cell cytotoxicity (P<0.05). Tumor volume showed the most correlation with IFN-γ. 

***H&E Staining***

Pathological evidence of lower vaccine efficacy including muscle extension, lymphovascular invasion, and perineural invasion was mainly seen in the control and CCSC vaccine groups. On the other hand, evidence of greater vaccine efficacy such as tumor necrosis, fibrosis, and inflammatory reaction was more common in HT-29 and SW-480 vaccine groups. Therefore, the pathological evidence also indicates that HT-29 and SW-48 vaccines are more effective than the CCSC vaccine (Table 2).

**Table 1:** Primer sequences for amplification of PGK1 and the genes examined

**Genes**	**Primer sequence 5′** **→** **3** **′**	**Amplicon size (bp)**
**PGK1**	F: CCACTGTGGCTTCTGGCATA R: ATGAGAGCTTTGGTTCCCCG	166
**ALDH1A1 **	F: GATCCCCGTGGCGTACTATG R: TGGATCTTGTCAGCCCAACC	202
**EPCAM**	F: GTGCTGGTGTGTGAACACTG R: GAAGTGCAGTCCGCAAACTT	155
**SOX2**	F: AACAGCCCGGACCGCGTCAA R: TCGCAGCCGCTTAGCCTCGT	189
**CD44**	F: TCCAACACCTCCCAGTATGACA R: GGCAGGTCTGTGACTGATGTACA	83
**CD133**	F: CACTACCAAGGACAAGGCGT R: TCCAACGCCTCTTTGGTCTC	153
**MYC**	F: AGCGACTCTGAGGAGGAACAAG R: TGGGCTGTGAGGAGGTTTGC	135
**CTNNB1**	F: CAACTAAACAGGAAGGGATGGAAGG R: CAGATGACGAAGAGCACAGATGG	239
**LGR5**	F: CCTTCCAACCTCAGCGTCTT R: AGGGATTGAAGGCTTCGCAA	248
**CDH1**	F: ATTCTGATTCTGCTGCTCTTG R: AGTCCTGGTCCTCTTCTCC	136
**CDH2**	F: ATGGTGTATGCCGTGAGAAG R: TGTGCTTACTGAATTGTCTTGG	196

PGK1: phosphoglycerate kinase 1, ALDH1A1: Aldehyde dehydrogenase 1 family member A1, EPCAM: Epithelial cell adhesion molecule, SOX2: sex-determining region Y-box 2, LGR5: Leucine-rich repeat-containing G-protein coupled receptor 5, CDH1: Cadherin-1, CDH2: Cadherin-2

**Table 2:** Results of pathological examination of tumor tissues removed from vaccinated mice

Tumor grade	Fibrosis	Calcification	Inflammatory reaction	Tumoral necrosis	Perineural invasion	Lymphovascular invasion	Muscle extension	Tumor size (mm^3^)	Mice numbers	Vaccine groups
**High**	-	-	+1	-	+	+	+	488	1	**Ctrl**
**High**	-	-	+1	-	+	+	+	515	2	
**High**	-	-	+2	-	+	+	+	499	3	
**High**	Multi- Focal	-	+3	Multi- Focal	-	-	-	15	1	**HT-29 vaccine**
**High**	-	-	+4	Extensive	-	-	-	18	2	
**High**	Multi- Focal	-	+3	Multi- Focal	-	-	-	20	3	
**High**	-	-	+2	-	+	+	-	88	1	**CCSC vaccine**
**High**	-	-	+2	-	+	+	+	94	2	
**High**	-	-	+2	-	+	+	+	36	3	
**High**	Multi-Focal	-	+3	Multi- Focal	-	-	-	21	1	**SW-480 vaccine**
**High**	Multi- Focal	-	+4	Extensive	-	-	-	9	2	
**High**	-	-	+2	-	+	+	-	22	3	

## Discussion

Vaccination is one of the most important subjects in cancer immunotherapy. Clinical trials have examined a variety of vaccines such as immunodominant peptides, pure antigens, naked DNA or recombinant viruse encoding tumor antigens, and whole tumor cells in cancer immunotherapy. Whole tumor cells usually express all TAAs and tumor-specific antigens (TSA), both known and unknown, leading to simultaneous induction of CD8^+^ and CD4^+^ cells (21). In a meta-analysis of nearly 1800 patients, patients vaccinated with whole tumor vaccines showed a significantly higher objective response compared with patients vaccinated with tumor-specific antigens (22). However, the sourcing of autologous tumor cells is highly invasive and confined by the quality of the biopsy material. As we experienced, the success rate of stem cell isolation is low and development of autologous vaccines is not feasible for all patients. Due to chemotherapy, radiation therapy, and severe yeast contamination, we lost six tumor samples out of seven (success rate of 14%). Besides, proliferation and characterization of slow-growing CCSCs were challenging and highly time-consuming, leading to delays in treatment and inefficiency of this method. To circumvent these restrictions, a number of cell line-based vaccines were developed. Cell lines are stable and guarantee an endless source of tumor cells. Also, allogeneic cell line-based vaccines are more recognizable for the patient’s immune system than autologous vaccines, reducing concern about their carcinogenicity and adjuvants’ necessity. However, they may lack the patient’s unique antigens. Recent studies showed that tumor antigens due to somatic mutations or epigenetic deregulations are rarely similar in different tumors and are highly heterogeneous, warranting the autologous whole tumor vaccine approach (4, 23). However, many studies have shown that allogeneic antigens can be a good alternative to autologous antigens. Allogeneic lysates from cell lines were used in dendritic cell (DC) therapy in various cancers and efficacy and safety of this approach were shown in mice and humans (24-26). Although methods such as DC therapy or adoptive T cell therapy are widely studied in clinical trials, these methods also require isolation of the patient’s immune cells or complex cellular and molecular processes that are time-consuming and costly (27-30). Whole tumor cell vaccines are a very simple and immediate method of vaccination without the need for DCs. GVAX approach was successful in preclinical trials and induced anti-tumor immunity in lymphoma, melanoma, prostate, renal, lung, and colon cancers (9). In an effort to design a general, cost-effective, and efficient CRC vaccine, we examined cell line-based vaccines in mice as well as comparing them with a prepared CCSC vaccine. Our results showed that the allogeneic SW-480 vaccine could effectively induce antitumor immunity in nude mice. All CCSC, allogeneic, and autologous vaccines significantly increased the serum level of TGF-β in mice. Despite differences in genetic content and chromosomal number, HT-29 and SW-480 demonstrated similar and considerable immunogenicity even without adjuvants. Therefore, the CRC vaccine could also be developed with other CRC cell lines, including SW-480 and HT-29. Unlike other clone vaccines such as GVAX, we did not use any adjuvants, but the vaccines could effectively reduce tumorigenesis in mice. On the other hand, the anticancer efficacy of the CCSC vaccine was less than that of the cell line-based vaccines. Unlike spleen weight, NK cytotoxicity, and TGF-β, IFN-γ did not show a significant increase in the CCSC vaccine group. IFN-γ had the most correlation with the tumor size and it was significantly increased in CRC cell line-based vaccine groups. IFN-γ is a cytokine that plays a critical role in both innate and adaptive immunity and functions as a stimulator of NK cells and neutrophils and the primary activator of macrophages (31). Thus, it seems that IFN-γ is more effective than TGF-β in inhibiting tumorigenesis. The lower efficacy of the CCSC vaccine in this study could be due to the inactivation of CCSC antigens during vaccine inactivation or lower antigen storage than CRC cell lines or other reasons. Cancer cells can also have different immunogenic potentials. Therefore, the CCSC vaccine group should be repeated with other patients’ CCSCs. What is clear, however, is that despite the effort and cost of developing the CCSC vaccine, available tumor cell lines provide efficient immunogenicity against CRC and lead to tumor inhibition in nude mice. Even if the CCSC vaccine showed greater immunogenicity, CCSC identification, isolation, proliferation, and inactivation are very challenging, and vaccine preparation could fail at any of these steps. Tumor heterogeneity and cancer cell plasticity have also made CCSC markers a challenge in the first step of the vaccine preparation process, i.e., CCSC isolation. We did not observe expression of LGR5 and CD133 in the isolated CCSCs, but they were CD44^+^. Several studies have reported LGR5 as a CCSC marker in CRC (32, 33). However, Kim *et al*. found no significant relationship between LGR5 and CRC in clinicopathological factors (34), and LGR5 has been reported as a tumor suppressor (35). As previously reported, CD133 knockdown did not affect proliferation, migration, invasion, and colony formation in CRC cell lines (36) and the number of CD133^+^ cells in spherical culture of CRC tumors could be between 2 and 96% (37). Therefore, the proposed markers for CCSC isolation may not be valid for all CCSCs. Overall, CCSC isolation is more useful in detecting tumor antigens and examining the tumorigenesis process than in developing autologous vaccines.

## Conclusion

Cell line-based vaccines have considerable advantages over CCSC vaccines in availability, ease and speed of preparation, and the possibility of multiple vaccinations. Conversely, isolating CCSCs and developing autologous CCSC vaccines are highly challenging. The present preclinical study showed that allogeneic vaccines developed from HT-29 and SW-480 can be an efficient and cost-effective option in the development of CRC vaccines. Future research on the efficacy and safety of these vaccines in humans is suggested. 

## Authors’ Contributions

FR Conceptualization, methodology, software, analysis, investigation, resources, writing; EN, FN, and SH Investigation; AA and MA Surgical tumor resection; NS and BM Pathological examination; MM FF and MG Methodology and resources; MRA Resources, conceptualization, and supervision.

## Funding

This work was supported by Mashhad University of Medical Sciences [Grant number 941474]; and Iran National Science Foundation [Grant number 96015793].

## Ethics Approval

The study (Code: 941474) was approved by the Human Research Ethics Committee of Mashhad University of Medical Sciences (Ethical approval codes: IR.MUMS.REC.1394.702) as well as the Institutional Animal Care and Use Committee of Mashhad University of Medical Sciences. 

## Coflicts of Interest

The authors declare that they have no conflicts of interest.
